# Expression of *Drosophila* VDRC GD RNAi lines in larval skeletal muscle often leads to abnormal proteostasis.

**DOI:** 10.17912/micropub.biology.000904

**Published:** 2023-08-04

**Authors:** Myung-Jun Kim, Michael B. O'Connor

**Affiliations:** 1 Department of Genetics, Cell Biology and Development, University of Minnesota, Minneapolis, Minnesota, United States of America

## Abstract

In *Drosophila* , multiple transgenic RNAi libraries have been generated to facilitate large-scale genetic screens *in vivo* . Although those libraries have helped generate many new discoveries, certain libraries are associated with technical drawbacks requiring caution in interpreting the results. Here, we report an unexpected effect of VDRC GD lines on proteostasis. When expressed in the larval skeletal muscle, 17 out of 20 GD lines induced protein aggregates enriched around the myonuclei while VDRC KK or TRiP counterparts had no effect. By contrast, the same GD lines failed to induce protein aggregates when expressed in the epidermal cells. Because the GD lines tested in this study target diverse classes of molecules and since the KK or TRiP counterparts exhibited no effect, we conclude that VDRC GD lines, for unknown reasons, tend to interfere with proteostasis in a tissue-specific and target-independent manner.

**Figure 1. Effect of VDRC GD lines on proteostasis in the larval body wall:**
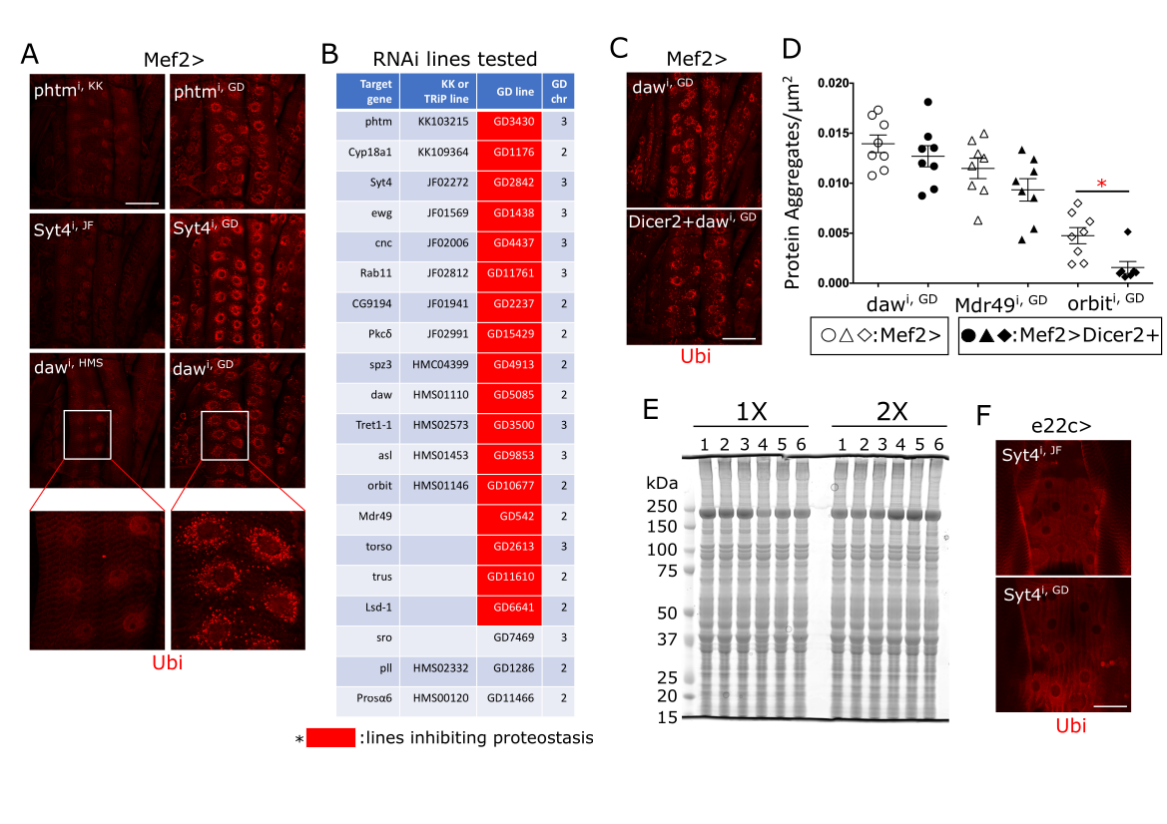
(A) Representative images of Ubiquitin staining on skeletal muscle. GD lines of *phtm* , *Syt4* and *daw* as well as KK or TRiP counterparts were expressed in the larval body wall muscle using Mef2-Gal4 driver and protein aggregates were examined with anti-Ubiquitin antibody. As shown, GD lines induce protein aggregates while KK or TRiP counterparts fail to do that. The bottom panels are enlarged images of similar area in the muscle expressing *daw* HMS or GD line. (B) Twenty GD lines in total were tested and the results are summarized here with a red shade highlighting the lines that induced protein aggregates in the muscle. At least 3 animals were examined per RNAi line. (C) Representative images of Ubiquitin staining on the muscle expressing *daw ^i, GD^* alone or together with *Dicer2* . (D) Quantification shows no increase in the number of protein aggregates per unit area by co-expression of *Dicer2* , demonstrating that processing of long dsRNA into siRNA does not enhance the protein aggregate formation. *P<0.05 from unpaired t-test. (E) Total protein staining on the gel. Lanes: 1- *Mef2>+* , 2- *Mef2>Mdr49 ^i^* ^, GD^ , 3- *Mef2>CG9194 ^i, HMS^* , 4- *Mef2>CG9194 ^i, GD^* , 5- *Mef2>orbit ^i, HMS^* , 6- *Mef2>orbit ^i, GD^* . The 1X and 2X denote two independent samplings. No ectopic protein band was induced by GD line expression. (F) e22c-Gal4 driver was used to express GD and TRiP lines in the epidermis. Representative images from *Syt4 ^i, HMS^* and *Syt4 ^i, GD^* expression show no protein aggregates. At least 3 animals were examined per RNAi line. Scale bars: 100 μm in (A and C) and 50 μm in (F).

## Description

Since introduced as a tool for selective gene silencing, RNA interference (RNAi) has played a widespread role in functional genetic screens in cultured cells as well as *in vivo* tissues (Schnorrer et al., 2010; Agrawal et al., 2013; Umer et al., 2019; Graca et al., 2021) . In *Drosophila* , *in vivo* screens became possible owing to transgenic RNAi libraries generated by multiple organizations including GD and KK lines from Vienna Drosophila Resource Center (VDRC), Transgenic RNAi Project (TRiP) lines from Harvard Medical School and National Institute of Genetics (NIG) RNAi strains from a Japanese consortium.

Despite offering an efficient way to knockdown the expression of target genes in a genome wide manner, the RNAi system is known to have certain technical limitations. The best known limitation is the off-target effects seen with short interfering RNA (siRNA) which reduces the expression of gene(s) other than the one that it was designed to target (Jackson et al., 2003) . Off-target silencing is proposed to occur when siRNA acts like a miRNA if there is a matching seed sequence in the UTR of the non-target gene’s mRNA (Saxena et al., 2003; Birmingham et al., 2006; Alemán et al., 2007). Because the off-target effect arises from the intrinsic property of siRNA that can act as miRNA in certain circumstances, it is associated with all the RNAi libraries. Besides the off-target effect, each RNAi library could also have its own unique problem originating from the way in which each RNAi library is constructed. For example, it has been shown that approximately 25% of VDRC KK lines lead to artifactual enhancement of the Hippo pathway independently of the intended target gene knockdown (Vissers et al., 2016) . The enhancement is due to ectopic expression of the *tiptop* gene which harbors the landing site for the KK RNAi transgenes in its 5’UTR. On the other hand, the pVALIUM10 vector-based TRiP lines contain attB sequences in their products because the RNAi transgenes are constructed using the Gateway system (Stanković et al., 2023). Therefore, if any pVALIUM10-based TRiP line is used with other transgenes also generated via the Gateway system, the transgenes will be targeted regardless of the intended target of the TRiP line. Taken together, these observations indicate that caution should be applied when interpreting the results obtained using RNAi lines and one needs to be open to the possibility that unidentified side effects may be associated with a specific RNAi library.

While working with VDRC GD lines over many years, we realized that these lines tend to induce protein aggregates in the larval skeletal muscle while TRiP or KK counterparts targeting the same genes had no effect. To examine this issue in a more systemic way, we compared the protein aggregate-inducing ability of all the GD lines available in our laboratory to that of TRiP or KK counterparts. When stained with anti-Ubiquitin antibody, skeletal muscles expressing *phtm ^i, KK^* , *Syt4 ^i, JF^* or *daw ^i, HMS^* lines showed no accumulation of protein aggregates ( Fig. 1A ). By contrast, GD lines targeting the same genes induced protein aggregates ( Fig. 1A ). The protein aggregates are enriched around the myonuclei but are also found in other cytoplasmic areas ( Fig. 1A, bottom panel ). We examined a total of 20 GD lines and 15 of them were compared with KK or TRiP counterparts. The results are summarized in Fig. 1B with a red shade indicating lines that interfere with proteostasis . As shown, 17 of 20 GD lines induced protein aggregates while none of the KK or TRiP counterparts examined did. The target genes of GD lines that inhibit proteostasis cover various classes of molecules including ion channel ( *CG9194* ), components in the ecdysone pathway ( *phtm* and *Cyp18a1* ), a secreted signaling molecule ( *daw* ), transcription factors ( *ewg* and *cnc* ) and a xenobiotic transporter ( *Mdr49* ). Others are involved in vesicle trafficking ( *Rab11* and *Syt4* ), microtubule organization ( *orbit* and *asl* ), signal transduction ( *torso* and *PKCδ* ), immune response ( *spz3* ) and nutrient regulation ( *tret1-1* and *Lsd-1* ). Because it seems unlikely that all of these molecules with such diverse functions converge to regulate proteostasis and also because KK or TRiP counterparts failed to induce protein aggregates, we believe that GD lines interfere with proteostasis through a mechanism other than specific inhibition of the target genes. In addition, GD lines are designed to be randomly inserted on the chromosomes (Dietzl et al., 2007) . Accordingly, approximately half of the GD lines examined in this study are inserted on 2 ^nd^ chromosome and the rest are on 3 ^rd^ ( Fig. 1B ) implying that the inhibition of proteostasis by GD lines is not likely to be an insertional effect.

VDRC GD lines encode long double-stranded RNAs (dsRNAs) which are processed into siRNAs by the action of Dicer. Because of this feature, overexpressing *Dicer2* has become a well-established practice in *Drosophila* to increase the RNAi efficacy of the dsRNA (Dietzl et al., 2007) . To examine if processing of long dsRNA into siRNA enhances protein aggregate formation, we chose 3 GD lines and expressed them with and without *Dicer2* . As presented in Fig. 1C and D, co -expression of *Dicer2* did not enhance protein aggregate formation, but actually decreased protein aggregates in the case of *orbit ^i, GD^* . The finding that increasing RNAi efficacy of GD lines via *Dicer2* co-expression did not lead to increased protein aggregates provides an additional line of evidence that the effect of GD lines on proteostasis does not rely on depletion of the target proteins. Next, we speculated that perhaps GD lines encode an ectopic peptide unrelated to the inverted target sequences and the putative ectopic peptide may disrupt proteostasis. To examine this possibility, we expressed some of the proteostasis-inhibiting GD lines in the muscle using the Mef2-GAL4 driver and compared the protein bands on the gel side-by-side to those of the Mef2-GAL4 driver alone or TRiP counterpart samples. As shown in Fig. 1E, we did not observe any ectopic band in GD line -expressing samples in total protein staining on the gel. This result suggests either that an ectopic peptide is expressed at a level below detection by this method, or that the GD lines interfere with proteostasis through a mechanism other than producing an ectopic peptide. Lastly, we overexpressed GD lines in the epidermis using the e22c-GAL4 driver to determine if GD lines interfere with proteostasis in a tissue other than muscle. As illustrated by representative images in Fig. 1F, GD lines failed to induce protein aggregates in the epidermis . Therefore, it appears that the proteostasis-interfering effect of GD lines does not occur in all tissues. Overall, one should exercise caution when using VDRC GD RNAi lines for examining proteostasis in muscles and perhaps other tissues. Since alterations in proteostasis are a hallmark of aging, this is an especially important consideration for researchers using GD-generated RNAi knockdown in *Drosophila* aging studies. It is still not clear to us how this artifact arises. Since the ubiquitin proteasome system (UPS) and autophagy are known to work together to suppress protein aggregate formation (reviewed in Johnston and Samant, 2021), the mechanism of GD line action could be targeting either or both of these systems. Therefore, future study of this interesting effect employing additional GD lines might uncover a novel mechanism for regulating proteostasis through one of these important molecular processes for regulating cellular homeostasis.

## Methods

Fly husbandry and stocks

Fly stocks were kept in vials containing standard cornmeal-yeast-agar medium. The larvae were raised on the same medium at 25 ^o^ C. RNAi lines used in this study are listed in Fig. 1B . Other lines are Mef2-GAL4 (BDSC 50742), e22c-GAL4 (BDSC 1973) and UAS-Dicer2 (BDSC 24650).

Antibody staining and imaging

Wandering larvae were dissected in Ca2 ^+^ -free HL3 solution and fixed in 3.7% paraformaldehyde (Electron Microscopy Sciences) for 40 min at room temperature. Fixed fillets were washed in 1X PBS and permeabilized in 1X PBT (0.5% BSA plus 0.2% Triton X-100 in 1X PBS) for 1 hour. The fillets were then incubated overnight at 4 ^o^ C with anti-Ubiquitin antibody (FK2, 1:200, Enzo Life Sciences) that detects mono- and polyubiquitinated proteins, followed by incubation with Alexa Flour 555-conjugated secondary antibody (1:200, Molecular Probes). Images were taken using an LSM 710 confocal microscope (Zeiss) with a 20X 0.8 N/A objective for images in Fig. 1A and C, or a 40X 1.20 N/A water objective for those in Fig. 1E . A 561 nm laser was used for excitation. Protein aggregates were counted using ‘Analyze Particles’ function in ImageJ software (NIH) and normalized to the muscle area.

Total protein staining

Four larval body wall tissues per sample were homogenized in 21 μl of RIPA buffer (Sigma, #R0278) supplemented with protease inhibitor cocktail (Complete mini, Roche) and incubated at 4 ^o^ C for 40 min with agitation. The samples were then centrifuged and 14 μl of supernatant from each sample was taken and mixed with 7 μl of 3X loading buffer. Samples were then incubated at 95 ^o^ C for 5 min to denature the proteins. Equal volumes from each sample were loaded onto 4-12% Bis-Tris gel (Invitrogen, NP0323BOX). After gel running, the proteins on the gel were visualized by staining with SimplyBlue SafeStain dye (Invitrogen, LC6060). The protein bands on the gel were imaged using Odyssey M imaging system (LI-COR Biosciences).

## References

[R1] Agrawal T, Sadaf S, Hasan G (2013). A genetic RNAi screen for IP₃/Ca²⁺ coupled GPCRs in Drosophila identifies the PdfR as a regulator of insect flight.. PLoS Genet.

[R2] Alemán LM, Doench J, Sharp PA (2007). Comparison of siRNA-induced off-target RNA and protein effects.. RNA.

[R3] Birmingham A, Anderson EM, Reynolds A, Ilsley-Tyree D, Leake D, Fedorov Y, Baskerville S, Maksimova E, Robinson K, Karpilow J, Marshall WS, Khvorova A (2006). 3' UTR seed matches, but not overall identity, are associated with RNAi off-targets.. Nat Methods.

[R4] Dietzl G, Chen D, Schnorrer F, Su KC, Barinova Y, Fellner M, Gasser B, Kinsey K, Oppel S, Scheiblauer S, Couto A, Marra V, Keleman K, Dickson BJ (2007). A genome-wide transgenic RNAi library for conditional gene inactivation in Drosophila.. Nature.

[R5] Graca FA, Sheffield N, Puppa M, Finkelstein D, Hunt LC, Demontis F (2021). A large-scale transgenic RNAi screen identifies transcription factors that modulate myofiber size in Drosophila.. PLoS Genet.

[R6] Jackson AL, Bartz SR, Schelter J, Kobayashi SV, Burchard J, Mao M, Li B, Cavet G, Linsley PS (2003). Expression profiling reveals off-target gene regulation by RNAi.. Nat Biotechnol.

[R7] Johnston HE, Samant RS (2020). Alternative systems for misfolded protein clearance: life beyond the proteasome.. FEBS J.

[R8] Saxena S, Jónsson ZO, Dutta A (2003). Small RNAs with imperfect match to endogenous mRNA repress translation. Implications for off-target activity of small inhibitory RNA in mammalian cells.. J Biol Chem.

[R9] Schnorrer F, Schönbauer C, Langer CC, Dietzl G, Novatchkova M, Schernhuber K, Fellner M, Azaryan A, Radolf M, Stark A, Keleman K, Dickson BJ (2010). Systematic genetic analysis of muscle morphogenesis and function in Drosophila.. Nature.

[R10] Stanković D, Csordás G, Uhlirova M (2022). Drosophila pVALIUM10 TRiP RNAi lines cause undesired silencing of Gateway-based transgenes.. Life Sci Alliance.

[R11] Umer Z, Akhtar J, Khan MHF, Shaheen N, Haseeb MA, Mazhar K, Mithani A, Anwar S, Tariq M (2019). Genome-wide RNAi screen in Drosophila reveals Enok as a novel trithorax group regulator.. Epigenetics Chromatin.

[R12] Vissers JH, Manning SA, Kulkarni A, Harvey KF (2016). A Drosophila RNAi library modulates Hippo pathway-dependent tissue growth.. Nat Commun.

